# On multiplexing in physical random number generation, and conserved total entropy content

**DOI:** 10.1038/s41598-023-35130-7

**Published:** 2023-05-16

**Authors:** Frederic Monet, Raman Kashyap

**Affiliations:** 1 Fabulas Laboratory, Engineering Physics Department, Polytechnique Montreal, 2900 Blvd Edouard-Montpetit, Montreal, H3T 1J4 Canada; 2 Poly-Grames, Electrical Engineering Department, Polytechnique Montreal, 2900 Blvd Edouard-Montpetit, Montreal, H3T 1J4 Canada

**Keywords:** Fibre optics and optical communications, Fibre lasers, Nonlinear optics, Supercontinuum generation

## Abstract

In the current article, we use a random supercontinuum based on a random Raman distributed feedback laser to investigate the generation of random numbers by spectrally demultiplexing the broad supercontinuum spectrum in parallel channels. By tuning the spectral separation between two independent channels, we test the most typically used statistical tests’ abilities to identify the required minimum spectral separation between channels, especially after the use of post-processing steps. Out of all the tests that were investigated, the cross-correlation across channels using the raw data appears to be the most robust. We also demonstrate that the use of post-processing steps, either least significant bits extraction or exclusive-OR operations, hinders the ability of these tests to detect the existing correlations. As such, performing these tests on post-processed data, often reported in literature, is insufficient to properly establish the independence of two parallel channels. We therefore present a methodology, which may be used to confirm the true randomness of parallel random number generation schemes. Finally, we demonstrate that, while tuning a single channel’s bandwidth can modify its potential randomness output, it also affects the number of available channels, such that the total random number generation bitrate is conserved.

## Introduction

Random number generation (RNG) is increasingly in demand for numerous applications, such as Monte Carlo simulations^1^, machine learning algorithms^2^ and secure communications^3^. While pseudo-random number generators based on algorithmic computations used to be sufficient for this purpose, certain applications requiring a very large amount of random numbers start revealing their limitations. As such, true random numbers generated by physical processes, as opposed to deterministic algorithms, have greatly risen in interest in recent years. Indeed, since they are based on true physical random systems, they do not suffer from the same reproducibility and periodicity issues that even the best pseudo-RNG systems exhibit. However, to ensure that the numbers generated are truly random, it is important to properly identify the origin of the randomness and quantify its potential. RNG based on quantum processes offer absolute certainty over the true randomness of the system, as the randomness originates from inherent quantum probabilities. However, the bitrates that can be achieved by these systems is relatively low, typically in the Mbps to low Gbps speeds^4^. This is insufficient for the applications described earlier, which consume random bits at staggeringly high speeds. Therefore, new sources of randomness have been investigated to generate random bits at higher rates than what is currently achievable with quantum processes.

The current state of the art relies on the entropy generated by semiconductor lasers operating chaotically due to external feedback to the cavity. Owing to the large bandwidths of chaotic lasers, RNG rates of hundreds of Gbps have been demonstrated^5^, and recent work demonstrated how these randomly generated bits can be extracted using all-optical quantization, which allows to overcome the limited bandwidths of electronic components like photodiodes and analog-to-digital converters^6^. However, the source of randomness in these chaotic systems is not as obvious as in the case of quantum systems and, in the race to achieve the largest possible RNG rate, many shortcuts have been taken. One of the most prevalent ones in the literature is the use of complex post-processing steps to hide existing correlations in bit sequences that are not sufficiently random to pass statistical testing^7–11^. A common post-processing step relies on the application of an exclusive-OR (XOR) operation between the original bit stream and a time-delayed version of it^7–9^. Even more complex post-processing operations, such as the use of successive numerical derivatives, offer the promise of generating more random bits per measurement than the original digitization used, which again should raise some concerns about the true randomness of the bit sequences thus generated^10,11^. In 2017, Hart et al*.* issued some recommendations for the evaluation of the entropy content of physical RNG systems^12^. In their article, they recommend that researchers should rely solely on minimally post-processed data for the purpose of generating true RNG, and that any bit sequence that requires the use of complex post-processing to pass statistical tests should be viewed as nothing more than a high-quality pseudo-random bit sequence. Furthermore, they argue that the physical origin of the entropy should be investigated and theoretically computed, rather than solely relying on statistical testing.

It can be observed that, since the publication of this article, these recommendations are increasingly being followed by researchers in literature. However, in order to continue publishing record-breaking RNG rates, multiplexing in RNG is now being investigated, where multiple channels simultaneously generate random bits in parallel^9,13–16^. This allows higher RNG rates by multiplying a single channel’s output by the number of parallel channels. Furthermore, since each channel generates its own RNG throughput, this type of system is perfectly well suited for parallel computing applications, which are increasingly used as they are more efficient, but require independent RNG streams^17,18^. One such way to achieve this is by using two or more chaotic laser sources and combining them in multiple ways to realize independent parallel channels using deterministic algorithms such as addition, subtraction or XOR operations for instance^9,16^. Recently, by combining the waveforms of three chaotic semiconductor lasers, and multiplexing the RNG across seven distinct channels, RNG rates as high as 2.24 Tbps were reported in literature^14^. Alternatively, the output of a single laser source could be separated either spectrally^13,19,20^, spatially^15^ or by polarization^21^ to generate random bits in parallel. Recently, Kim et al. reported an astounding 250 Tbps rate achieved by a single laser diode through the interaction of multiple lasing modes in a specially tailored cavity^15^. However, as Hart et al. recommendations applied for single channel RNG^12^, at the moment there is no real consensus how to translate these for multiplexed RNG systems. It is the goal of this article to help provide insights how these recommendations might be formulated, by studying a novel RNG architecture and identifying the conditions ensuring independence between the multiplexed channels.

In our recent work, we demonstrated how a single channel narrow linewidth random Raman distributed feedback fiber laser could generate a theoretical entropy content of 540 Gbps^22^. We showed that, as illustrated by Hart et al.^12^, the evaluation of this theoretical entropy content was critical, as a bit sequence generated at 1.28 Tbps (more than twice the theoretical entropy content) passed all the National Institute of Standards and Technology (NIST) statistical tests^23^. This highlighted the need for theoretical entropy content estimation for proper true RNG characterization. By performing minor modifications to the laser cavity architecture described in that article, the output characteristics can be significantly changed to result in the generation of a random Raman supercontinuum, induced by modulation instability and Raman self-frequency shift. Using this approach, we demonstrate how this laser can be used for multiplexed random generation by spectrally sampling multiple channels from this supercontinuum. In the current proof of concept, the spectral multiplexing is achieved using a pair of fiber Bragg gratings (FBGs), instead of using a typical arrayed waveguide grating (AWG) for instance^20^, which can provide a much higher number of spectral channels. However, our unconventional technique allows us to tune the spectral separation between the two channels to determine the minimum separation required ensuring they are indeed uncorrelated. Furthermore, we test some of the techniques found in literature to measure correlations between channels and demonstrate that caution should be exercised when performing these tests to ensure they truly measure what they are expected to, especially when using post-processed data.

## Single-channel random number generation

### Random supercontinuum generation

**Figure 1:**
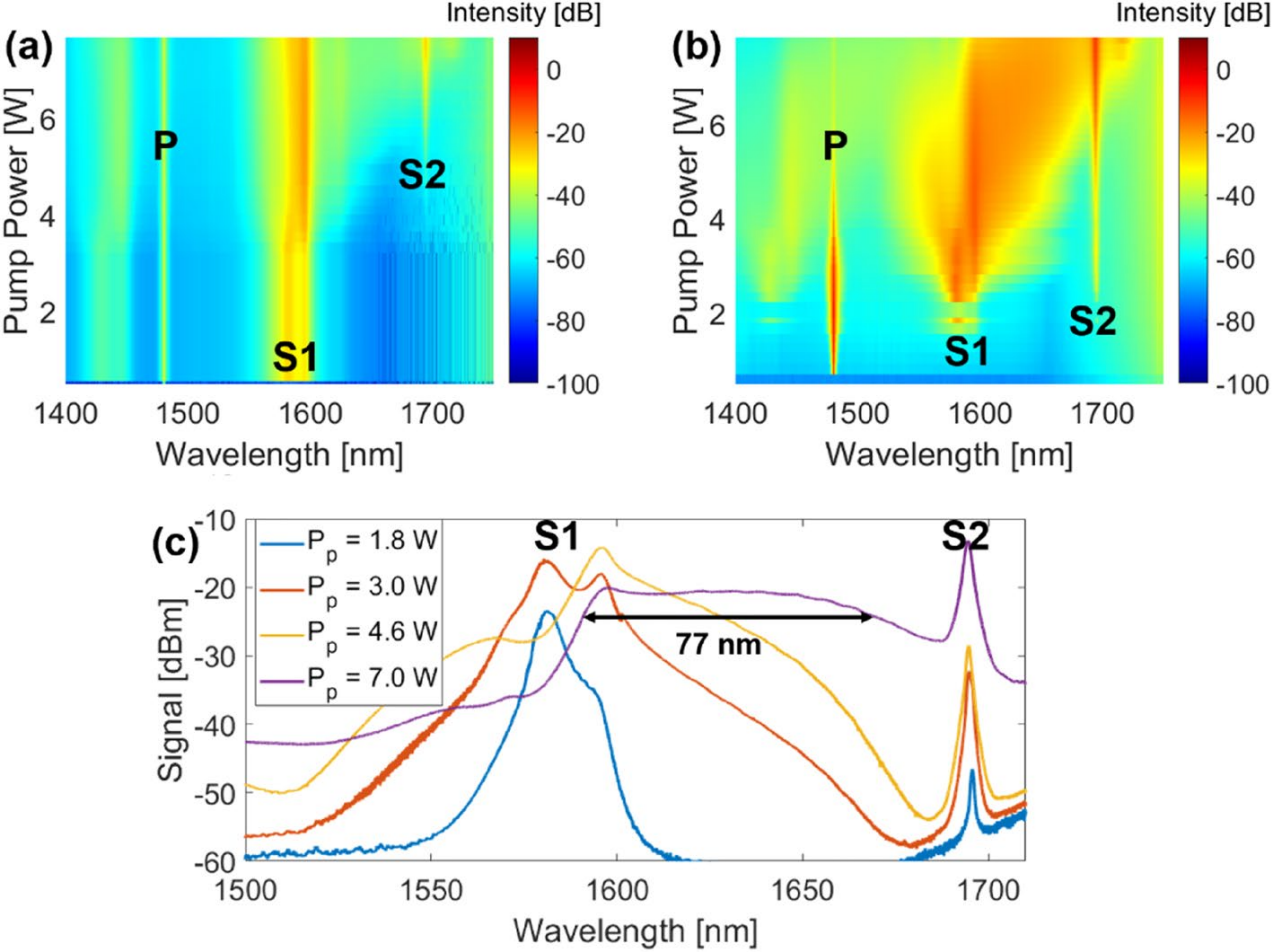
Output spectra of the random laser, both in (**a**) backward direction and (**b**) forward direction. At high pump power, the random supercontinuum can be observed. The pump (P), first Stokes (S1) and second Stokes (S2) lines are identified on both spectra. The spectra of the forward direction output at a few pump powers is also displayed in a 2D plot for better visualization in (**c**). The 77 nm bandwidth (measured at − 3 dB) is also identified.

In our previous work, we fabricated a random Raman laser closed on one side with a 100 mm long phase-controlled apodized FBG, relying on Rayleigh backscatter on the other end to provide random feedback, while also providing Raman gain, thus achieving laser action^22^. We show here that, by replacing the FBG with the Fresnel reflection of a fiber end tip, lasing can also occur, with a significantly simpler cavity design. Furthermore, since the Fresnel reflection is broadband, lasing is no longer constrained to the FBG’s wavelength, which fixed the lasing wavelength in our previous work. Indeed, as we show in Fig. 1, while lasing is initially achieved at a wavelength of 1580 nm (which corresponds to the maximum of the Raman gain generated by the 1480 nm pump), a second peak is then generated at 1595 nm, and eventually becomes dominant. This two peak structure is typical of distributed feedback random lasers, and has been observed in multiple instances^24^.

At higher pump powers, the spectrum starts broadening, and shifts towards the longer wavelengths. This is attributed to Raman intra-pulse scattering, which is due to the fact that the lasing wavelength is slightly above the zero dispersion wavelength (ZDW), placing this laser in the normal dispersion regime. Near the ZDW, nonlinear effects such as modulation instability (MI) can break the laser output into multiple ultrashort, random pulses, which will then experience Raman intra-pulse scattering, shifting them to longer wavelengths. In our previous work, MI side-lobes were observed near the lasing wavelength, however this is our first observation of self-frequency shift, which further confirms our previous observations. At even higher pump powers, the second Raman Stokes line starts becoming apparent, and eventually dominates the forward output. As the pump power increases, the self-frequency shift starts spanning the entire band between the first and second Raman Stokes peaks, covering a bandwidth of 77 nm within a 3 dB flatness. This bandwidth is only limited by the available pump power and would extend further in the longer wavelengths at higher pump powers. This type of behavior has been observed in other random laser architectures^25,26^, although generally with much more complex setups requiring one or more FBGs, and typically with at least two different types of fiber, whereas our setup relies simply on the Fresnel reflection of the fiber tip, and one bundle of optical fiber.

### Single-channel random number generation

**Figure 2:**
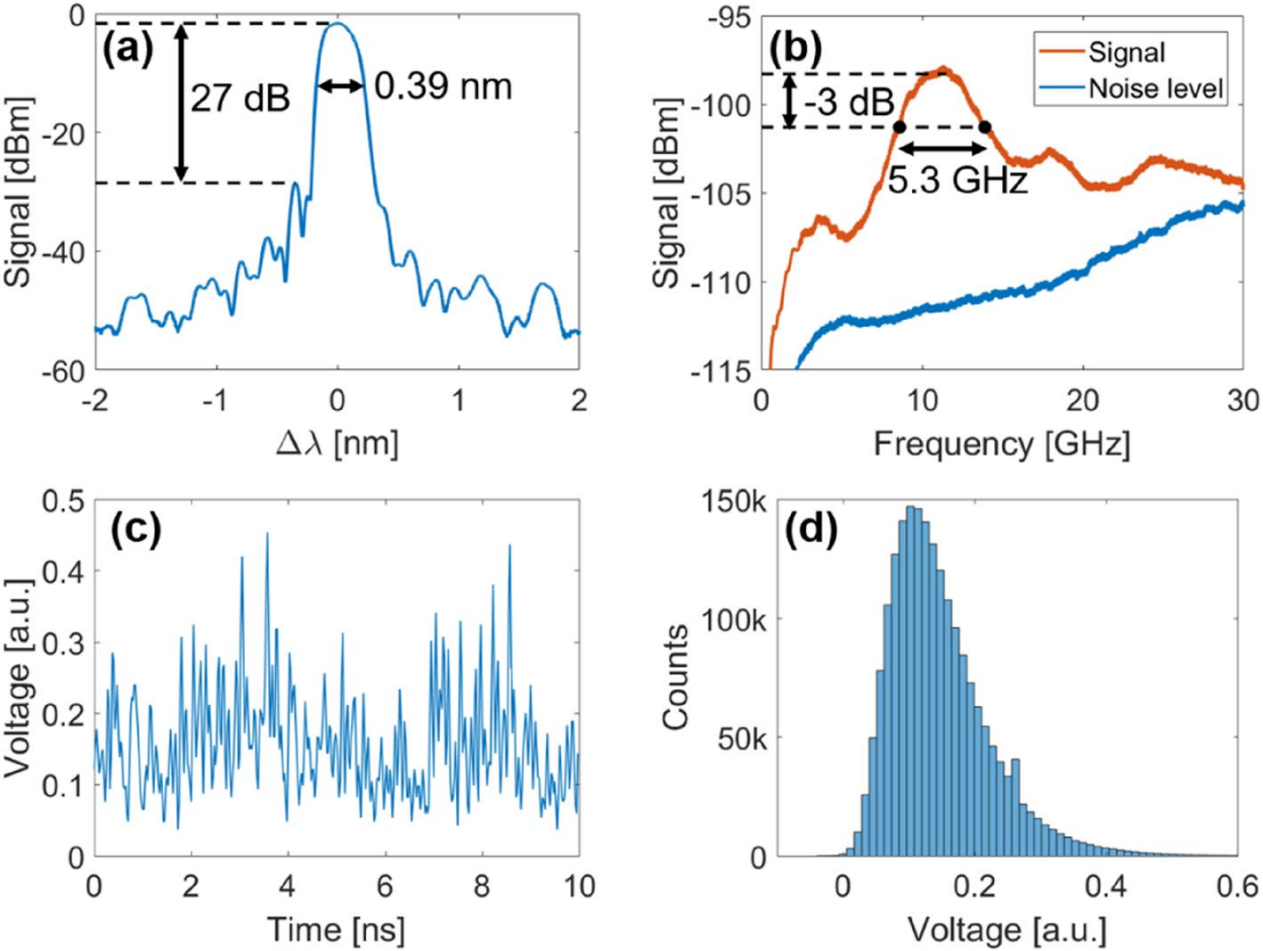
(**a**) Single-channel’s optical spectrum, displaying the narrow − 10 dB bandwidth of 0.39 nm. (**b**) RF spectrum of the single channel, displaying a 5.3 GHz bandwidth at − 3 dB. (**c**) Sample time sequence, measured at 40 GSa/s. (**d**) Histogram of the channel output, sampled over 2 million points.

Before attempting multiplexed RNG operation, the ability of a single channel to generate random bits was investigated and quantified. From the supercontinuum output, a single 0.39 nm channel was isolated using an apodized 9-mm long FBG (see “Methods” Section for more details). Figure 2a displays the resulting channel’s output spectrum, where the isolation with respect to the supercontinuum can be observed, with an extinction ratio of 27 dB.

In order to use this channel to generate random bits, the output of the channel was first converted to the electrical domain by a high-speed 70 GHz photodiode, and the electrical signal thus generated was digitized by an 8-bit 12 GHz ADC, sampled at 40 GSa/s. From this digital signal, the 3 least significant bits (LSBs) were extracted, resulting in a random bit sequence generated at 120 Gbps. The bit sequence was then analyzed by the National Institute of Standards and Technology (NIST) statistical testing suite^23^, using 1000 samples of 1 Mb each. The results are presented in Table 1 below. As can be seen, the bit sequence passes all of the statistical tests at the *p* < 0.01 significance level, since all tests have at least 0.980 success rate, and the lowest *p*-value is above 0.0001.

**Table 1:** Results of the NIST SP 800–22 for the random bit sequence generated at 120 Gbps.

Statistical test	*p*-value	Proportion
Frequency	0.011383	0.991
BlockFrequency	0.753844	0.991
CumulativeSums	0.204439	0.992
Runs	0.155499	0.985
LongestRun	0.753844	0.987
Rank	0.792508	0.993
FFT	0.054314	0.995
NonOverlappingTemplate	0.011144	0.981
OverlappingTemplate	0.316052	0.992
Universal	0.030197	0.987
ApproximateEntropy	0.031428	0.989
RandomExcursions	0.003797	0.984
RandomExcursionsVariant	0.002406	0.981
Serial	0.110734	0.987
LinearComplexity	0.844641	0.992

### Physical entropy evaluation

In order to properly quantify the randomness of an RNG scheme, it is important to evaluate the physical entropy generated by such a system. Indeed, as noted by Hart et al*.*^12^, randomness evaluation through statistical tests, such as is customary in RNG literature, is not sufficient to properly ascertain the physical randomness of one RNG scheme. Indeed, they demonstrated that multiple RNG schemes in literature claim an RNG bitrate larger than the underlying physical entropy generated by the systems they used, making them pseudo-random numbers at best. We also arrived at similar conclusions in our previous work^22^.

The maximum entropy of a given system is given by

1 $$h_{0} = \min (\tau^{ - 1} ,2\Delta f)(N_{\epsilon } - D_{KL} (p(x)||u(x)))$$

where *τ*^*−*1^ is the sampling rate, *Δf* is the limiting bandwidth, $N_{\epsilon }$ is the number of bits used in the digitization, *p*(*x*) is the probability density function (PDF) of the entropy source, *u*(*x*) is the PDF of the uniform distribution over the same interval as *p*(*x*) and *D*_*KL*_ is the Kullback–Leibler divergence from *u*(*x*) to *p*(*x*), in bits^27^. Indeed, the ideal distribution for RNG purposes is a uniform distribution, so that each number has an equal probability of being generated. However, since physical entropy sources rarely follow a uniform distribution, the correction factor *D*_*KL*_ must be applied to the maximum theoretical entropy due to the divergence between the ideal uniform distribution and the actual entropy source’s distribution.

In order to characterize the theoretical physical entropy generated by such a channel, its output was analyzed by an electrical signal analyzer (*Agilent* PXA N9030A) which can measure up to 50 GHz. The electrical spectrum associated to the isolated channel is shown in Fig. 2b. The electrical bandwidth, which limits the channel’s physical entropy, was defined at the − 3 dB bandwidth, and was measured at 5.3 GHz. A sample time sequence of the channel’s output, sampled at 40 GSa/s, is displayed in Fig. 2c, while a histogram of the signal’s distribution is shown in Fig. 2d. From this histogram, the Kullback–Leibler divergence can be computed at 1.38 bit. Using Eq. (1), the theoretical entropy content can thus be estimated to be 70 Gbps. Rather unsurprisingly, the computed theoretical entropy content is smaller than the one that was experimentally demonstrated. This highlights again the insufficiency of the NIST statistical tests to distinguish between true random numbers and high-quality pseudo-random bits. For the rest of this manuscript, we will consider that this channel has the potential for 70 Gbps random bit generation, even though the sample generated at 120 Gbps passed all tests.

## Parallel RNG

The previous section demonstrated our ability to generate random bits in a single-channel scheme by isolating a narrow spectral channel from the generated supercontinuum. However, to take full advantage of the broad supercontinuum bandwidth, multiple channels could be used to generate random bits in parallel. A naive computation of the number of potential spectral channels would be to divide the supercontinuum’s 77 nm bandwidth by the channel’s 0.4 nm bandwidth, resulting in a total of 192 parallel channels. From the 70 Gbps single-channel demonstrated in the previous section, this would result in a total potential bitrate up to 13.44 Tbps. If the entropy content had not been evaluated, using the 120 Gbps rate that was validated with the NIST statistical tests, this would push the bitrate even higher, at 23.04 Tbps, although these would theoretically only be high-quality pseudo-random bits. However, one key assumption made here is that the signals generated by two separate spectral channels are independent. To maximize this independence, we used apodized FBGs to eliminate the side-lobes that could contribute to crosstalk across the channels. However, it is not obvious that the channels are completely uncorrelated since they originate from the same laser, even though they come from different parts of the spectrum. To investigate this further, two identical apodized FBGs were used to generate two parallel channels. Since the two FBGs are identical and centered at the same wavelength, the two channels are expected to be perfectly correlated. Then, by applying strain to one of the FBGs, its central wavelength can be shifted by up to 15 nm (corresponding to a 1% strain). This allows the observation of how the wavelength separation between the two channels influences the correlations between the channels.

In literature, two tests are mainly used to quantify these correlations across parallel channels: cross-correlations and mutual information. Both these tests will be performed on the extracted sequences, and the impact of post-processing will be quantified. One key advantage of our technique is that it allows the use of a reference, which corresponds to the case where the two FBGs are unstrained. Performing these tests on this reference allows us to ensure that the post-processing steps have no effect on the validity of the tests. Indeed, if the post-processing steps decrease the minimum required spectral separation, but also decrease the ability of the test to identify the existing correlations when the channels are expected to be correlated, then these post-processing steps actually reduce the ability of the statistical test to perform as intended.

### Cross-correlations across channels

**Figure 3:**
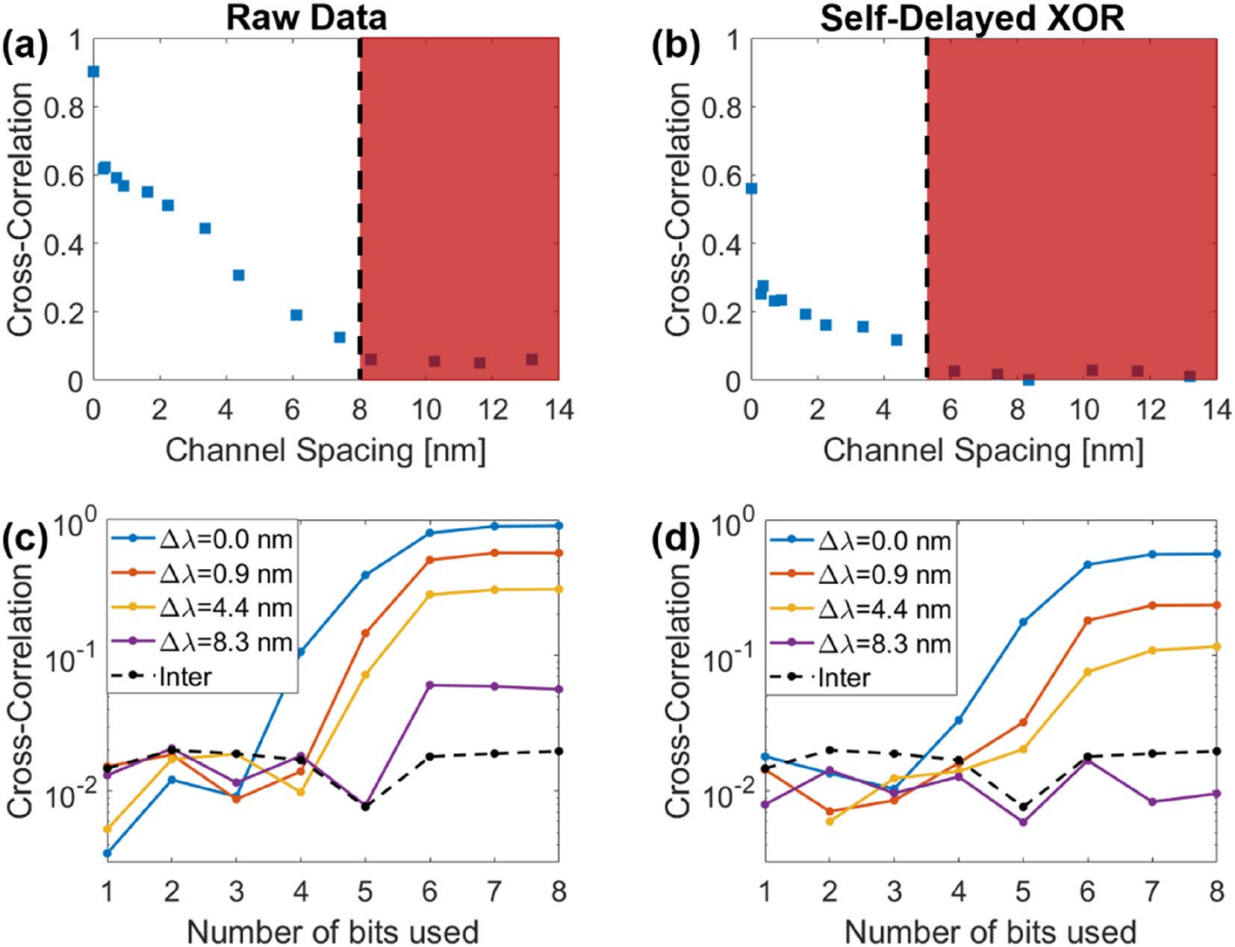
Cross-correlation values using (**a**) the raw data and (**b**) the same data after applying a self-delayed XOR operation, as a function of the channels’ spectral spacing. The region highlighted in red corresponds to spectral separations considered uncorrelated. The cross-correlation is also computed after extraction of a number of LSBs, using (**c**) the raw data and (**d**) the same data after applying a self-delayed XOR operation. The *inter* values (dashed line) displays the cross-correlation measured between two separate measurements as comparison.

The first statistical test that was applied to the extracted data was the computation of the cross-correlation across the two channels, as a function of their spectral separation. To correct for the delay caused by any mismatch between the two channels’ propagation length, the cross-correlation was computed between the two channels, and the delay *τ*_*m*_ associated with the maximum of this cross-correlation function was thus identified as the mismatch between the two channels’ lengths. The cross-correlation between the two intensities *X* and *Y* is given by

2 $$C_{X,Y} (\tau ) = \frac{{\int {\delta X(t + \tau )\delta Y(t)} dt}}{{\sqrt {\int {\left( {\delta X(t)} \right)^{2} dt\int {\left( {\delta Y(t)} \right)^{2} dt} } } }},$$

where $\delta X\left( t \right) = X\left( t \right) - \left\langle {X(t)} \right\rangle$. These results are illustrated in Fig. 3a. As can be observed, when the two FBGs’ central wavelengths are matched, a correlation of almost 1 is measured (0.90), which confirms that the signals are the same on both channels. As the spectral separation increases due to the applied strain, the correlation continually decreases. It remains above 0.5 (-3 dB) up to a channel separation of 2 nm, and the signals remain correlated until a spectral separation of 8 nm. This has a dramatic effect on the total achievable RNG rate, as this limits the number of potential parallel channels to only 11, rather than the 192 obtained by our initial naïve computation.

This approach is significantly different from what is typically observed in literature. While certain authors do present the cross-correlation computations based on the raw data^13,28^, it is far from the norm. Indeed, when this analysis is performed, most authors rather perform it on post-processed data^20^, usually after extracting the random bits^14,19^, sometimes followed by further post-processing steps such as XOR operations^15,16,29^. To compare the results, the same process was applied with the current data. For example, a typically used post-processing step is the self-delayed XOR operation. Even though Hart et al. argued against the use of such post-processing techniques^12^, they are still used in recent publications, both in single-channel^30,31^ and multiplexed RNG schemes^13–15,21^. To quantify the impact of the self-delayed XOR operation, we digitized the raw data using 8-bit representation (the digitization used by the real-time oscilloscope used in these experiments). An XOR operation was then applied between the resulting bit sequence and a version of itself delayed by 1.6 ns. After performing this additional post-processing step, an important decrease in correlations can be observed (see Fig. 3b). Indeed, as the spectral separation is increased, no correlation can be observed past 5 nm. While it may be tempting to claim that the use of the self-delayed XOR increased the number of potential multiplexed channels due to this reduction in the required spectral spacing, it should be noted that it also decreased the correlations when there was no spectral separation between the channels. Indeed, the correlation between the two signals in this case dropped from 0.90 to 0.56. The fact that it had such a drastic effect even with no spectral separation rather implies this operation reduced the ability of the cross-correlation test to accurately detect the existing correlations.

Another approach typically used in literature is to first extract LSBs before characterizing channel independence. To reproduce this data processing, a varying number of LSB was extracted from the digitized raw data and the cross-correlation between the two channels was computed after LSBs extraction. These results are presented in Fig. 3c. Indeed, as can be observed, extracting the LSBs before computing the cross-correlation greatly decreases the correlations between the channels, and it appears that when 3 LSBs or less are extracted, the correlations across channels are similar to those obtained from two separate measurements (the *inter* case displayed with the dashed line). This would seem to indicate that LSBs extraction improves the independence across channels. However, here again, the proposed methodology allows the computations of this correlation when there is no spectral separation between the two channels, which is not typically measured in literature. This leads to the observation that the correlations also disappear when a small enough number of LSBs is selected, even when the two channels are expected to be perfectly correlated. This would seem to imply that no spectral separation is required to ensure that the channels are completely uncorrelated, which obviously does not make sense. Our measurements show that, while the correlations measured after LSBs extraction are indeed lower, they cannot be relied upon to quantify the channels’ independence. Instead, the full raw data should be used to compute the correlations across channels, in a similar fashion to what we have shown in Fig. 3a. Similarly, when looking at the effect of the self-delayed XOR after LSB extraction in Fig. 3d, it can be observed that the correlations converge to that of the *inter* case even faster than without this post-processing operation. Again, one could use this as evidence that the self-delayed XOR, combined with the LSBs extraction, enhances the channels’ independence, but based on the above arguments, we showed that both these techniques only decrease the effectiveness of the cross-correlation test.

### Mutual information between channels

The second statistical test that was realized was the computation of the mutual information between the channels. Mutual information between two bit-streams *X* and *Y* is defined as

3 $$I(X;Y) = \sum\limits_{y \in Y} {\sum\limits_{x \in X} {P_{(X,Y)} (x,y)\log_{2} \left( {\frac{{P_{(X,Y)} (x,y)}}{{P_{X} (x)P_{Y} (y)}}} \right)} } ,$$

where *P*_*X*_(*x*) is the probability that *X* = *x*, *P*_*Y*_(*y*) is the probability that *Y* = *y* and *P*_*(X,Y)*_(*x*,*y*) is the probability that (*X*,*Y*) = (*x*, *y*). The base-2 logarithm is chosen here to express the mutual information in bits. The computation of the mutual information across channels is another statistical test used in literature^15,19^, because from it one can infer whether the bits generated in one channel contain information on the bits in the other channel. Obviously, for RNG purposes, this mutual information should be as close to zero as possible.

**Figure 4:**
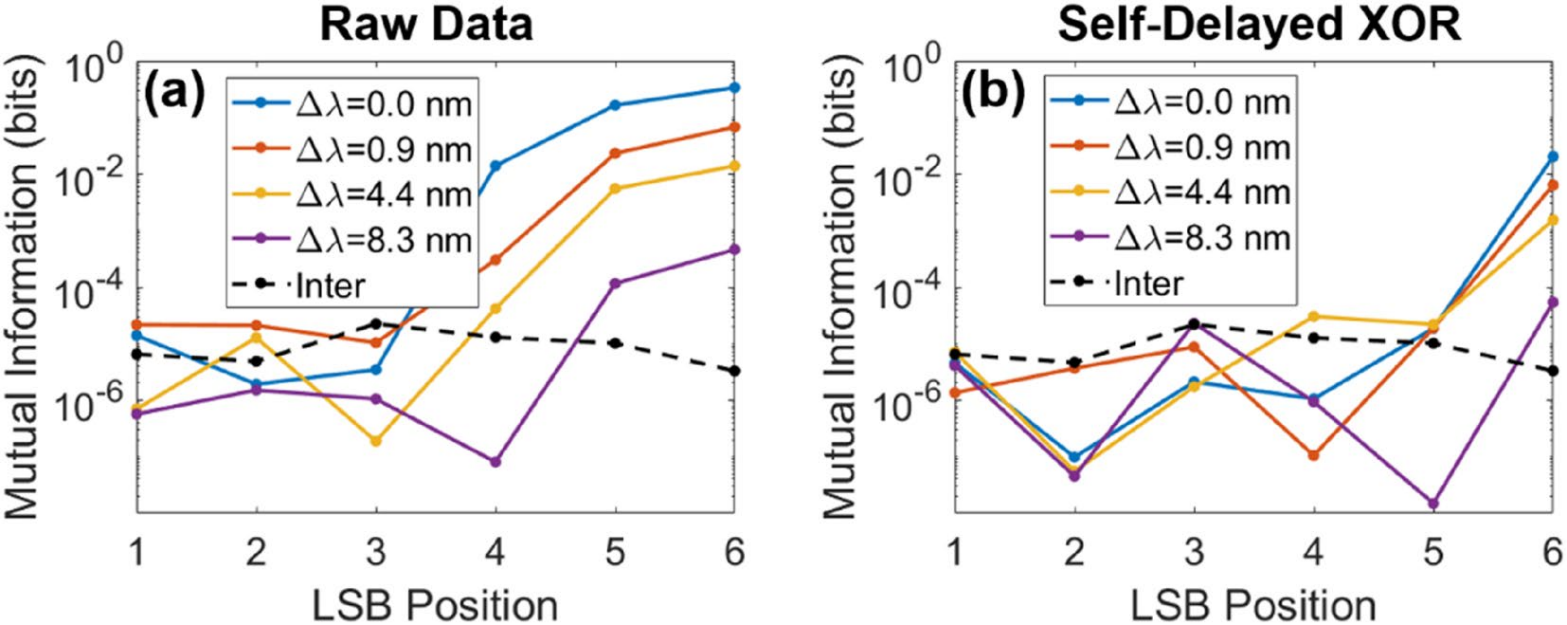
Mutual information between the two channels, as a function of the LSB position, where 1 is the least significant bit and 8 the most significant one, for various spectral separations. The mutual information is computed from (**a**) the digitized raw data and (**b**) after performing a self-delayed XOR operation. In both cases, the sequence was digitized using 8 bits, and consisted of 10^5^ samples. The sequence length is inversely proportional to the mutual information limit of detection, observed in the *inter case* as 10^−5^.

Here, the mutual information was computed for each bit position, for various spectral separations. The mutual information is only computed for the 6 LSBs, because the first two do not contain enough information due to their uneven distribution of ones and zeros (for more details, see Supplementary Information). As can be observed in Fig. 4a, the mutual information between the two channels for the most significant bits tested (LSB Position 6) is very high, and quickly decreases as the bits become less significant, until the last 3 LSBs, where the mutual information is of the same order of magnitude (~ 10^−5^) as the one obtained from two different measurements (here again named the *inter* measurement with the dashed line).

This behavior is more or less exactly the same than the one that was observed with the cross-correlation measurements. Here too, when the spectral separation is zero, even though the mutual information is much higher for the more significant bits, it also rapidly decreases and becomes undistinguishable from the *inter* case for the last 3 LSBs, which would again seem to indicate that no spectral separation is required to achieve independent multiplexed channels, as long as 3 LSBs or less are kept. For completeness, the same data treatment was realized after an additional self-delayed XOR operation was applied on the generated bits, where again the mutual information decreases much faster for all plotted spectral separations, as shown in Fig. 4b. What this demonstrates is that the mutual information computation is as vulnerable as the cross-correlation measurements to LSB extraction and therefore cannot be considered a robust method of ensuring independence across channels. However, looking at the mutual information of each bit individually (instead of only looking at the least significant ones), as depicted in Fig. 4, appears to be somewhat more robust, as the mutual information is much higher for the more significant bits (e.g. LSB 6 in Fig. 4). However, again, the use of the self-delayed XOR greatly decreases the mutual information content, even when there is no spectral separation. Therefore, it still appears that the cross-correlation computation using solely the raw data is the most robust technique to ensure independence across channels.

### Channel widths and conserved total entropy

**Figure 5:**
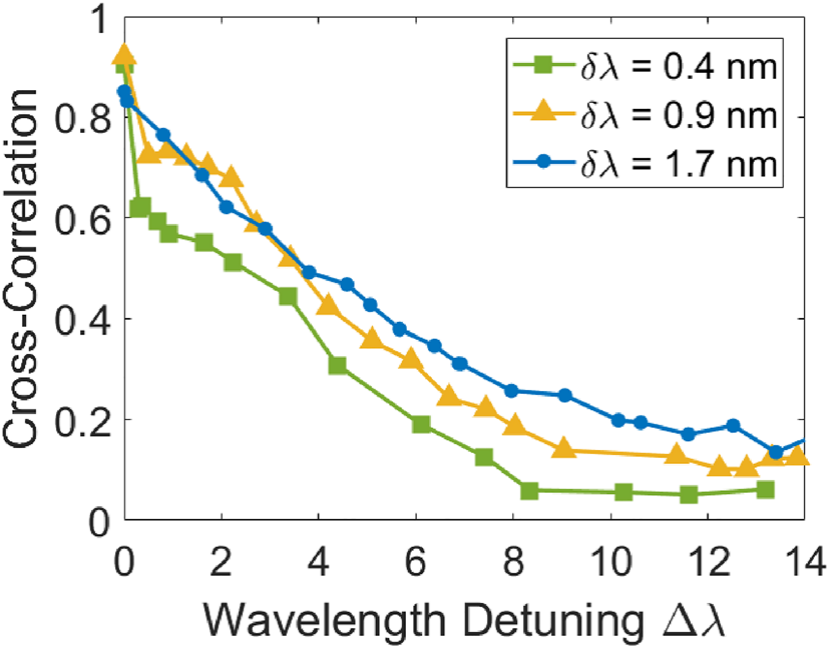
Cross-correlation values as a function of wavelength detuning, for different channel spectral widths.

From the analysis of the previous sections, it appears that, in order to ensure proper decorrelation between neighboring channels, the 0.4 nm channels must be spectrally separated by a minimum of 8 nm. Considering this, it is tempting to wonder whether the use of channels broader than the 0.4 nm demonstrated so far might help with the total achievable RNG rate. Indeed, it makes intuitive sense that a broader spectral channel might have the potential for higher single-channel RNG, and the current channel width is much narrower than the minimal channel spacing. Indeed, as can be observed in Table 2, when varying the channel’s bandwidth from 0.4 to 1.7 nm, a broadening of its electrical spectrum can be observed, which will result in increased RNG potential, as per Eq. (1). However, in order to make sure that the use of a broader channel’s bandwidth indeed results in a greater total RNG rate, the analysis performed in “Cross-correlations across channels” Section must be repeated for these new channel widths. These results are displayed in Fig. 5. As can be observed, while using a broader channel spectral width increases the potential for single-channel RNG generation, it also increases the minimum spectral separation between channels, which will decrease the number of potential channels that can be extracted from the supercontinuum. As such, the total RNG rate remains constant at around 760 Gbps, as can be observed from the data in Table 2, where an increase in single-channel RNG rate is accompanied by a decrease in the number of achievable channels. This is perhaps unsurprising, as the total RNG rate should be limited by the entropy generated by the laser, regardless of the number of channels it is separated into. However, in practice, single-channel RNG rates are limited by the speed of the electronics, and it can therefore be helpful to use several parallel channels generating bits at more reasonable rates. On the other hand, an increasing number of parallel channels increases complexity and costs. Therefore, depending on the entropy source that is used, proper optimization must thus be performed to perform RNG across the optimal number of channels based on these limitations.

**Table 2:** RNG Potential of different channel’s optical bandwidth δλ.

Optical bandwidth δλ (nm)	Electrical bandwidth Δf (GHz)	Single-channel RNG potential (Gbps)	Number of potential channels	Total RNG potential (Gbps)
0.4	5.3	70	11	772
0.9	6.4	85	9	763
1.7	8.2	109	7	760

## Discussion

Novel methods to generate high-speed physical RNG systems are increasingly popular in literature. However, in the race to report the highest RNG rate, one must exercise extreme caution that the process is indeed based on true physical randomness, and that the bits generated are not simply high-quality pseudo-random ones. Other researchers have formulated recommendations in that regard, for example cautioning against post-processing steps that can only hide existing correlations at best. However, there exist no such recommendations when generating random bits in parallel using multiplexed channels, which is becoming an increasingly popular subject of research. In this work, using a very simple random Raman laser architecture, we generated a 77 nm random supercontinuum that allowed us to investigate parallel RNG using spectrally multiplexed channels. It should be pointed out that only the region of the spectrum corresponding to the flat (within 3 dB) supercontinuum was considered for RNG. This allows the assumption that each channel produces the same entropy, as they each have approximately the same intensity, and they owe their origins to the same nonlinear optics effects. Even if the exact value of each of the individual channels might vary slightly, they are expected to be similar to the one calculated earlier, and therefore this should not significantly affect the total entropy numbers reported here. To obtain a larger number of RNG channels, the spectral components outside this bandwidth could be used. However, the assumption above would most likely no longer be valid. Additionally, this would require photodiodes with wider operating ranges, as the intensities vary wildly outside this flat bandwidth.

Furthermore, this technique allowed us to look at which tests can be used to ensure independence between the channels. We demonstrated that both cross-correlation and mutual information computations after LSBs extraction are insufficient to ensure that the channels are truly uncorrelated, as they both indicate that the tested channels are uncorrelated even when there is no spectral separation between them. From an initial assumption of 192 random channels in the supercontinuum, we showed that using the cross-correlation tests reduced the true randomness to only 11, demonstrating the pitfalls of a naïve approach. While the origins of these correlations were not investigated in this work, literature on this subject suggests that the most probable processes are pump-to-Stokes relative intensity noise transfer, cross-phase modulation and four-wave mixing effects^32,33^. A methodology such as the one suggested by Vatnik et al.^34^ could allow to identify which of these effects is mainly responsible for the observed correlations.

We also demonstrated that the use of an exclusive OR (XOR) operation on the extracted bits further decreased the calculated correlations. Rather than improving the randomness of the extracted bits, this post-processing step reduced the ability of the two investigated statistical tests to properly quantify the existing correlations. From our tests, simply computing the cross-correlations using the raw data appears to be the most robust method to determine the minimum spectral separation. To compute a more exact estimation of the total entropy content, as well as ensure each channel’s independence, this methodology should then be repeated over each individual channel, rather than only over a single one as demonstrated here. It is our opinion that, as more and more researchers investigate the potential of parallel RNG using multiplexed channels, more advanced statistical tests should be developed to ensure there are no correlations across the channels. We believe the methodology presented in this article can be used in conjunction with these tests to ensure they properly measure what they intend. Finally, while this technique was demonstrated in the case of spectral multiplexing, we believe it can be translated easily to RNG techniques relying on spatial multiplexing with minimal changes.

## Methods

**Figure 6:**
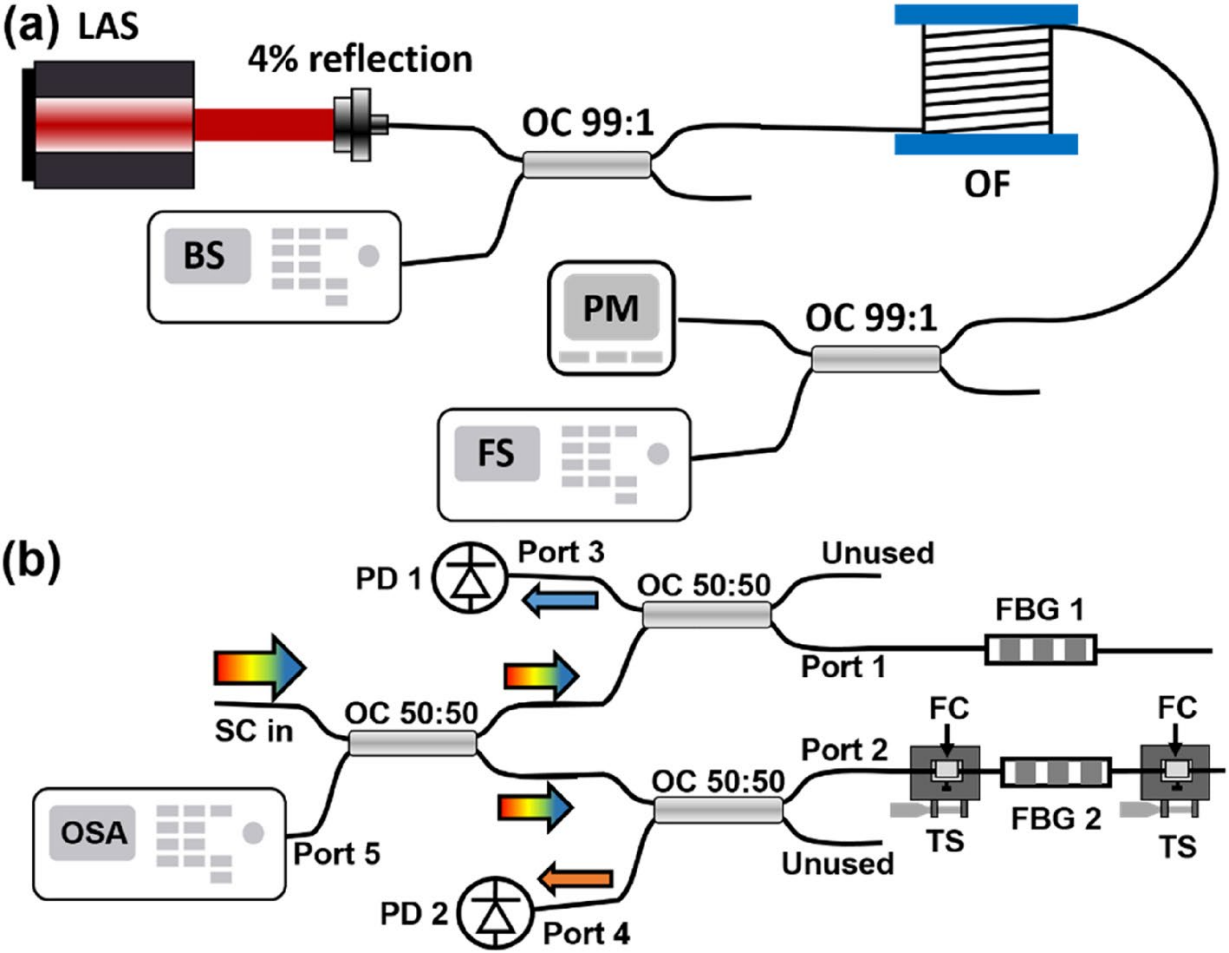
(**a**) Experimental setup for the random laser, with LAS the pump laser, OC the optical coupler, OF the optical fiber bundle, PM the power meter used to measure the output power, BS and FS, respectively measure the backward and forward spectra of the laser. (**b**) Experimental setup for the parallel channel demultiplexer. The supercontinuum SC is split in two channels by a 50:50 optical coupler OC, and each channel’s central wavelength is defined by its own FBG (Ports 1 and 2). The optical signal of each channel is converted to an electrical signal by a pair of high-speed photodiodes PD (Ports 3 and 4). The spectral separation between the channels can be tuned by applying strain on one FBG, using the translation stages TS, where on top of each one a fiber clamp FC (*FiberVice™*, *PhotoNova Inc*) holds the fiber. An optical spectrum analyzer OSA monitors the spectral separation between the two channels (Port 5).

The random Raman supercontinuum relies on a half-open random laser cavity architecture. The feedback of the cavity is provided on one end by the 4% Fresnel reflection at the tip of the fiber, which is cleaved at a 0° angle, and on the other side by the random Rayleigh backscatter of 6.66 km of non-zero dispersion-shifted (NZ-DS) single mode fiber (SMF-LS, Corning), while the gain is provided by stimulated Raman scattering. All fiber outputs are cleaved at a 4°-angle to prevent parasitic reflections. The NZ-DS fiber has a zero-dispersion wavelength (ZDW) near 1560 nm. This ZDW closely coincides with the first Raman Stokes peak (1580 nm) of the CW pump laser at 1480 nm that is injected into the fiber. The RF spectra of this laser was measured by a 50 GHz electrical spectrum analyzer, while the optical spectra (both backward and forward) were measured by connecting the output to an optical spectrum analyzer with a 0.01 nm resolution. The random laser cavity is displayed in Fig. 6a.

To simulate the multiplexing operation, the forward output of this laser was separated into two channels by a 50:50 coupler. Two identical 9 mm FBG were written with a cosine apodization profile by UV laser inscription, in a Talbot interferometer scheme, using a commercial semi-automatic FBG writing station (*BraggATune™*, *PhotoNova Inc*). This resulted in two identical channels with a 0.39 nm bandwidth centered at 1630 nm. This wavelength was selected as it is far beyond the Raman gain bandwidth, to ensure that the reflected channels do not affect the lasing operation. This was validated by monitoring the optical spectrum and laser output power before and after including this channel separator, showing no significant change. In future work, the reflections could be further prevented by the use of a high power isolator. By applying strain on one of the FBGs, the spectral spacing between the two channels could then be tuned to investigate the effects of this spacing on the correlation across the two channels. The two outputs were then connected to two 70 GHz photodiodes, and digitized by a 12 GHz, 8 bit analog-to-digital converter (ADC), real-time oscilloscope. The spectral separation between the two channels was monitored by an optical spectrum analyzer connected to the reverse output of the optical coupler separating the two channels, to measure the central wavelength of both channels simultaneously (Port 5 of Fig. 6b). Figure 6b displays the experimental setup used to demultiplex the two channels.

## Supplementary Information

Supplementary Information.

## Data Availability

All data used in this manuscript can be made available upon reasonable request to the authors.
