# Supplementary material for: On multiplexing in physical random number generation, and conserved total entropy content
Source: Sci Rep. 2023 May 16;13:7892. doi: 10.1038/s41598-023-35130-7 (PMC10188537; doi:10.1038/s41598-023-35130-7)
Supplement: Supplementary file 1 — Supplementary Information. [file 41598_2023_35130_MOESM1_ESM.docx]

Supplementary Material for “On Multiplexing in physical Random Number Generation, and conserved total entropy content”

Mutual Information for all 8 bits

In the experimental data, due to the digitization process of the real-time oscilloscope used, the probabilities of each bit of being either a “0” or a “1” was uneven, depending on the position of that bit. The probability of each bit being a “1” is illustrated in Fig. S1(a), in orange, as a function of its position. The most significant bit (LSB position 8) is almost always a 0 (*P_X_*(*x*=1) = 10^-4^), and thus contain very little information, while the 5 least significant bits (LSB positions 1 through 5) have an approximatively 50% chance of being either a 1 or a 0. This is due to the fact that the binning used by the oscilloscope is not perfectly centered with the probability density function (PDF) of the random signal, as well as the due to the slight asymmetry of the PDF. As such, the most significant bits will have an uneven distribution of “0”s and “1”s. This is not a problem for the RNG process demonstrated here, which only utilizes the 3 LSBs, but affects negatively the mutual information computation for these most significant bits, since it has *P_X_*(*x*) in the denominator. The result of the mutual information computation is shown in Fig. S1(a) in blue and compared with the probability *P_X_*(*x*=1) of X being a 1.


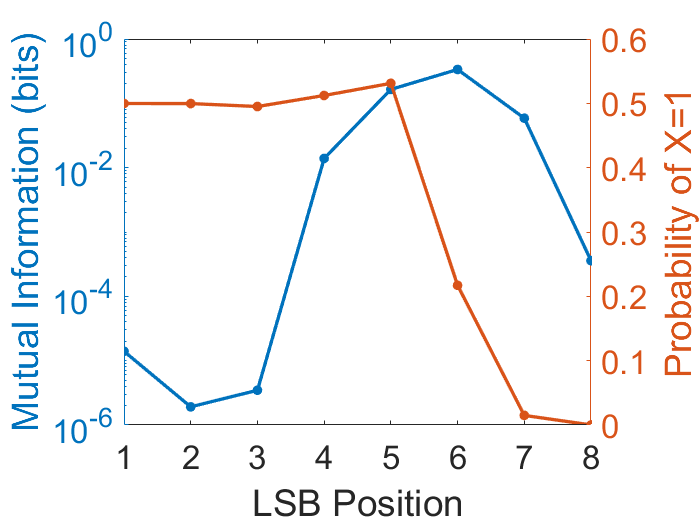


Fig. S1 (a) Mutual information for all LSB positions (blue), compared to that bit’s probability of being equal to 1 (orange), in the case where there was no spectral separation between the two channels. LSB position 1 is the least significant bit, and 8 is the most significant bit.

For demonstration purposes, this is schematized in Fig. S2 with a Gaussian distribution with a 4 bit digitization, where the distribution and its associated binning is shown in Fig. S2(a), and the resulting probabilities *P_X_*(*x*=1) for each LSB Position in displayed in Fig. S2(b). Again, it can be observed that the least significant bits have a probability *P_X_*(*x*) around 50%, whereas the most significant ones diverge from an even distribution.


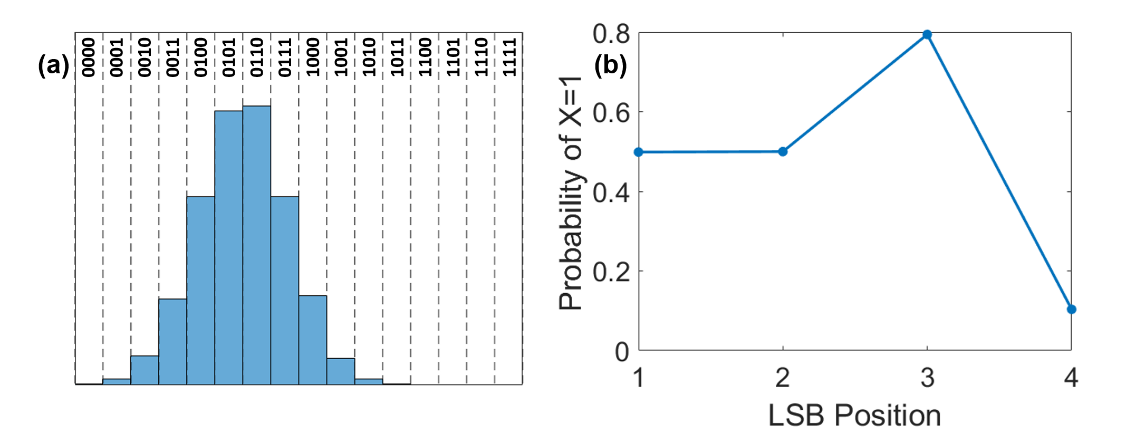


Fig. S2 (a) Histogram of a Gaussian distribution, binned using 4 bits. (b) Probability of all bits being equal to 1, depending on the LSB position.
